# Lipidomic profiling of amniotic fluid and its application in fetal lung maturity prediction

**DOI:** 10.1002/jcla.23109

**Published:** 2019-12-05

**Authors:** Zheng Cao, Jingrui Liu, Xin Xie, Sien Zhan, Wei Song, Shaowen Wu, Zheng Sun, Ying Dong, Guodong Tang, Yilin Liu, Lin Li, Min Shen, Yanhong Zhai, Jihua Zou, Xiaowei Liu

**Affiliations:** ^1^ Department of Laboratory Medicine Beijing Obstetrics and Gynecology Hospital Capital Medical University Beijing China; ^2^ Department of Obstetrics Beijing Obstetrics and Gynecology Hospital Capital Medical University Beijing China; ^3^ Operating Room Beijing Obstetrics and Gynecology Hospital Capital Medical University Beijing China; ^4^ Prenatal Diagnosis Center Beijing Haidian Maternal and Child Health Hospital Beijing China; ^5^ Beijing Omics Bio‐tech Co., Ltd Beijing China; ^6^ Central Laboratory Beijing Obstetrics and Gynecology Hospital Capital Medical University Beijing China; ^7^ Reference Laboratory Medical System Biotechnology Co., Ltd Ningbo China

**Keywords:** amniotic fluid, FLM, L/S ratio, LC‐HRMS, lipidomics

## Abstract

**Background:**

The pulmonary surfactant especially lipids in amniotic fluid can reflect the development stage of fetal lung maturity (FLM). However, the conventional lecithin/sphingomyelin (L/S) ratio method by thin layer chromatography (TLC) is insufficient and inconvenient for FLM prediction in clinical practice.

**Methods:**

The amniotic fluid samples were collected from the pregnant women in labor or undergoing amniocentesis and analyzed for its lipid contents with the liquid chromatography coupled with high‐resolution mass spectrometry (LC‐HRMS) method and the lamellar body count (LBC) method. To reveal the lipidomic profiling of different FLM stages, three groups of amniotic fluid samples including 8 from premature group (gestational week (GW) < 37), 10 from mature group (GW < 37), and 10 from mature group (GW > 38) were compared with the control group (n = 6) of 18 GWs separately.

**Results:**

In the FLM prediction study, the sensitivity of the LC‐HRMS method and LBC method was 91% and 73%, respectively; the specificity was 100% and 95%, respectively. The most significant metabolic pathway was linoleic acid metabolism between the premature group and the control group. Both glycerophospholipid metabolism and glycosylphosphatidylinositol‐anchor biosynthesis were enriched in the mature groups. In search of potential FLM prediction markers in amniotic fluid, 8 phosphatidylcholines, 1 sphingomyelin, and 1 phosphatidylethanolamine were significantly increased in the mature groups compared with the premature group.

**Conclusion:**

An efficient LC‐HRMS method for L/S ratio in predicting FLM was established. The linoleic acid metabolism may play an important role in the fetal lung development.

## 1 INTRODUCTION

Respiratory distress syndrome (RDS) is a major cause of morbidity and mortality in preterm infants. The deficiency of pulmonary surfactant resulting in lung prematurity is the most common cause of RDS in newborns.^1^ The incidence of newborn RDS increases with decreasing gestational age as the lungs are the final fetal organs to mature.^2^ Prediction for fetal lung maturity (FLM) plays an important role in the prevention of RDS in the preterm newborns. The conventional tests for FLM are based on the assessment of the amount of surfactant in the amniotic fluid, which results from the exchange of lipids between the developing lungs and the amniotic fluid.^3^ These conventional methods include measuring lecithin/sphingomyelin (L/S) ratio by thin layer chromatography (TLC), phosphatidylglycerol (PG), surfactant/albumin ratio (S/A), and lamellar body counts (LBC). However, the above methods have inevitable limitations and are not efficient or accurate enough to determine the FLM. The L/S ratio is the quantification of the lecithin and sphingomyelin on TLC after a series of extraction, dissolution, and separation of amniotic fluid samples. The L/S ratio rises with increasing gestational weeks as the sphingomyelin concentration remains relatively constant, while lecithin concentration increases during the late pregnancy.^3^ However, this method is time‐consuming, imprecise, and susceptible to blood and meconium interference.^4^ PG is the last lipid to increase in fetal lung surfactants, and there are two methods currently available to detect PG: the quantitative TLC and the qualitative agglutination. The main advantage of PG method is that it is not affected by blood and meconium contamination. However, its relatively high false‐positive rate presents a hurdle for wide application in clinical practice.^5^ The principle of the S/A method is based on the fact that the fluorescent polarization is high when the dye binds to albumin and low when the dye binds to the lung surfactants. This method was once widely used in clinical prediction of FLM. Unfortunately, the instrument and reagents of the S/A assay were discontinued since 2011 and the interference caused by blood and meconium cannot be ignored.^4^, ^5^ Lamellar bodies are secreted into the alveolar space from type II pneumocytes and are further transported into the amniotic cavity. Although the similar size between lamellar bodies and platelets makes the automated hematological cell counters suitable for LBC with its platelet channel, the lack of appropriate quality control reagents and universal threshold value limits its application.^4^, ^6^

Lipidomics is a newly emerged research focus which studies cellular lipids on a large scale using advanced analytical technological tools, such as mass spectrometry. Lipidomics aims to study the structures and the functions of the complete set of lipids in a specific cell or organism as well as their interactions with other cellular components to elucidate the pathways and networks in biological systems.^7^ As the existing laboratory methods of FLM are mostly based on the lipid components in amniotic fluid, we proposed that the lipidomics did not only encourage the establishment of an efficient L/S ratio method by liquid chromatography coupled with high‐resolution mass spectrometry (LC‐HRMS), but could also reveal the metabolic profiling difference in amniotic fluid between the fetal lung mature group and the premature group.

## 2 MATERIALS AND METHODS

### 2.1 Subjects

Thirty‐three amniotic fluid samples without blood or meconium contamination were collected from the pregnant women who were in labor or undergoing amniocentesis in Beijing Obstetrics and Gynecology Hospital from January 2018 to December 2018. They were used in the comparison between the LC‐HRMS‐based L/S ratio method and the in‐house LBC method.

Further, another 34 amniotic fluid samples were collected from pregnant women in labor of different gestational weeks (GWs) and were tested in the targeted lipidomics study. The FLM was judged by the Apgar scores of the newborns. The term “premature” referred to the newborns with penalty points for unsatisfactory or disturbed respiratory function, while the “mature” referred to the newborns with full Apgar scores. Of the 34 amniotic fluid samples, 6 samples were from 18 GWs, 8 premature samples from <37 GWs, 10 mature samples from <37 GWs, and 10 mature samples from >38 GWs.

### 2.2 Reagents and methods

#### 2.2.1 Chemicals

Methanol, acetonitrile, formic acid, isopropyl alcohol, trichloromethane, ammonium acetate, and water were purchased from Fisher Scientific (Thermo Fisher Scientific, Inc, Leicester, USA). Ammonium acetate was obtained from Sigma‐Aldrich (St. Louis, MO, USA). The internal standard mixtures including phosphatidylcholine (PC), phosphatidylethanolamine (PE), phosphatidylglycerol (PG), sphingomyelin (SM), and ceramide (Cer) were purchased from Avanti Polar Lipids (Alabama, USA). The purity of all the internal standards was higher than 99% (https://avantilipids.com), which met the requirement of MS quantification method.

#### 2.2.2 Amniotic fluid preparation

The amniotic fluid sample processing steps before the LC‐HRMS were described as follows. Briefly, 200 μL of amniotic fluid of each patient was mixed with 580 μL extraction solution of trichloromethane/methanol (3:1, v/v) and 20 μL internal standard of PC, PE, PG, SM, and Cer. After ultrasonic treatment for 1 hour, 100 μL water was added and mixed thoroughly. Then, 300 μL supernatant was transferred to an empty vial and dried with nitrogen gas followed by reconstitution in 200 μL of isopropanol/acetonitrile (1:1, v/v) and LC‐HRMS analysis.

#### 2.2.3 LC‐HRMS method

The Dionex™ UltiMate™ 3000 Rapid Separation LC system (Thermo Fisher Scientific, MA, USA) combined with an ACQUITY UPLC BEH C_8_ column (2.1 mm × 100 mm × 1.7 μm, Waters, Milford, MA, USA) was used for lipid separation. The mobile phases used in the above LC system consisted of solvent A (0.1% formic acid‐acetonitrile/water (3:2 v/v)) and solvent B (0.1% formic acid‐isopropanol/acetonitrile (9:1 v/v)) with a gradient elution program (0‐2 minutes, 0%‐30% B; 2‐12 minutes, 30%‐70% B; 12‐12.5 minutes, 70%‐95% B; 12.5‐13 minutes, 95%‐100% B; 13‐13.1 minutes, 100%‐0% B; 13.1‐15 minutes, 0%‐0% B). The flow rate of the mobile phase was 0.26 mL/min. The column temperature was maintained at 45°C. A Q Exactive™ Hybrid Quadrupole‐Orbitrap Mass Spectrometer (Thermo Fisher Scientific, MA, USA) with an ESI source was used to quantify each lipid with the full scan mode. The positive and negative HESI‐II spray voltages were 3.7 and 3.5 KV, respectively. The heated capillary temperature and the heated vaporizer temperature were 320°C and 300°C. Nitrogen was used as the sheath and the auxiliary gas with the pressure settings of 30 and 10 psi, respectively. The nitrogen collision gas was set at 1.5 mTorr. The parameter settings for the full mass scan mode were as follows: 70 000 of resolution and 100‐1500 of m/z range. The calibration was customized to the Q Exactive instrument to keep the mass tolerance <5 ppm. The system was controlled by XCalibur 2.2 software (Thermo Fisher Scientific, MA, USA).

In the L/S ratio determination experiments, the sum peak intensity of 64 lecithin and 23 peak intensity of sphingomyelin was used for the ratio calculation.

#### 2.2.4 Data processing

The targeted lipid library applied in the metabolomic study was based on our existing lipid library which contains 151 lipids with known structures, including 88 PCs, 27 PEs, 23 SMs, and 13 Cers. There had been examples employing the targeted lipidomic approach in studies of other diseases such as schizophrenia, diabetic cardiomyopathy, and clear cell renal cell carcinoma.^8^, ^9^, ^10^ The raw data from LC‐HRMS were imported to the Skyline software (Windsor, UK) for the relative quantification of the lipid species based on the retention time and the accurate mass in the constructed polar lipids database (in‐house database). The chromatographic data for each lipid were manually analyzed to determine the quality of the signal and peak shape. Before chemometrics analysis, all of the detected ion signals in each sample were normalized to the obtained total ion count value.

#### 2.2.5 LBC method

The 3 mL of amniotic fluid from each participant was inverted for 1 minute before centrifugation (276 × g) for 5 minutes. Then, the supernatant was detected for LBC on the platelet channel of SYSMEX XN‐2000/3000 automatic blood cell analyzer (Kobe, Japan) following the manufacturer's standard operation procedure for platelet counting.

### 2.3 Study design

#### 2.3.1 LC‐HRMS method validation

In the validation study for the L/S ratio determination, 20 μL of all the amniotic fluid (AF) samples was mixed evenly and used as the quality control in the following steps. Briefly, the pure methanol solution and subsequent quality controls were used to balance the chromatographic column. In the actual experiments, every 5 AF sample injections were immediately followed by a quality control sample. The average coefficient of variations (CVs) of PC and SM were calculated for the precision evaluation. The cluster of the quality control samples in the principal component analysis (PCA) score scatter plot was used to present the overall stability and repeatability of this lipidomic analysis.

#### 2.3.2 Comparison of L/S ratio method and the LBC method

The LC‐HRMS L/S ratio results and LBC results of 33 amniotic fluid samples (11 premature and 22 mature samples by Apgar scores) were compared. The clinical specificity and sensitivity were calculated for these two methods.

#### 2.3.3 Lipidomic profiling of amniotic fluid samples

As the 37 GWs is the cutoff used to define a preterm labor,^11^ and to explore the changings of lipids in amniotic fluid with the development of fetal lung, three groups of amniotic fluid samples (8 from preterm newborns (<37 GWs) with premature lungs, 10 from preterm newborns (<37 GWs) with mature lungs, and 10 from term newborns (>38 GWs) with mature lungs) were compared with the control group (n = 6) of 18 GWs separately. Their corresponding significant lipid metabolic pathways were analyzed.

#### 2.3.4 Lipid biomarkers for predicting FLM in amniotic fluid

The FLM mature amniotic fluid samples were grouped together and compared with the premature group to identify any potential lipid biomarkers for predicting FLM. In this study, *P* value of t test < 0.05, VIP > 1.1, and fold change > 2 were used to search for significantly different lipid metabolites.

### 2.4 Statistical analysis

For the pathway enrichment analysis, the *P* value was calculated by Holm‐Bonferroni method and the whole process was performed on the MetaboAnalyst 3.0 (http://www.metaboanalyst.ca). For the study of potential lipid biomarkers for predicting FLM, the *p* value was calculated by t test method and the whole process was performed on the SIMCA 14.1 software (Umetrics AB, Umea, Sweden).

## 3 RESULTS

### 3.1 Validation of the L/S ratio method by LC‐HRMS

**Figure 1:**
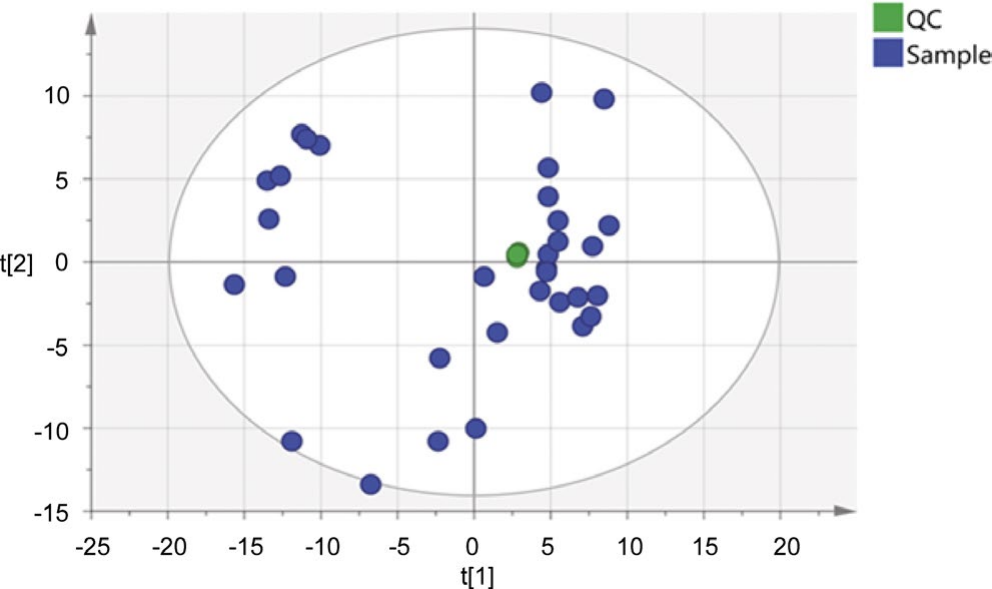
The principal component analysis (PCA) score plot of the 6 quality control (QC) and amniotic fluid samples. QC samples were applied to exhibit the stability of the LC‐HRMS system. The cluster of the QC samples in the PCA score scatter plot showed a satisfactory stability and repeatability of this lipidomic analysis approach. The colors display the subjects from different groups. The green dots represent QC samples, and the blue dots represent amniotic fluid samples

The L/S ratio assay by LC‐HRMS was a semi‐quantitative method calibrated with the internal standards that are exogenous compounds used to assess and adjust the stability of the analytical methods. The targeted lipids detected in this lipidomic analysis are mixtures of multiple lipid components, not a single compound, making it hard to perform routine recovery experiment for evaluation of accuracy. However, the internal standards in the LC‐HRMS method can correct the matrix effect of the AF samples and allow for evaluating the assay precision. As described in the Methods section, the quality control samples were repeated for 6 times to obtain the CV of each lipid. The average CV of 64 PC compounds and 23 SM compounds was calculated to be 3.8% and 3.4%, respectively. The cluster of the quality control samples in the PCA score scatter plot showed a satisfactory stability and repeatability of this lipidomic analysis approach (Figure 1).

### 3.2 Method comparison of the L/S ratio with the LC‐HRMS and the LBC

Of the 33 samples, 11 samples were premature and 22 samples were mature determined by Apgar scores. Ten out of the 11 premature samples and all the 22 mature samples were accurately predicted for FLM by the LC‐HRMS L/S ratio method using the cutoff value of 10.0 that was previously reported in a mass spectrometry FLM study.^12^ In comparison, with the cutoff value of <50 × 10^9^/L,^13^ the LBC method was able to make correct diagnosis for 8 of 11 premature samples and for 21 of 22 mature samples (Table 1). As a result, the sensitivity of LC‐HRMS L/S ratio method and LBC method was 91% and 73%, respectively; the specificity of LC‐HRMS L/S ratio method and LBC method was 100% and 95%, respectively (Table 2).

**Table 1:** The results of L/S ratio and LBC with 33 amniotic fluid samples

No.	L/S ratio^a^	LBC^b^ (×10^9^/L)	Sampling GW^c^	FLM^d^ outcome
1	1.84	1	18	—
2	1.50	2	18	—
3	1.57	2	18	—
4	1.94	2	18	—
5	1.30	2	18	—
6	2.27	1	18	—
7	1.71	2	18	—
8	5.05	75	32	Premature
9	1.99	48	35	Premature
10	2.13	56	36	Premature
11	15.24	65	36	Premature
12	33.92	57	39	Mature
13	45.71	57	38	Mature
14	99.69	58	37	Mature
15	63.11	56	36	Mature
16	96.00	53	35	Mature
17	57.81	55	38	Mature
18	41.36	55	37	Mature
19	49.58	52	37	Mature
20	61.14	57	39	Mature
21	26.76	58	36	Mature
22	54.35	48	39	Mature
23	58.40	56	39	Mature
24	55.85	56	37	Mature
25	107.17	60	39	Mature
26	65.10	60	37	Mature
27	112.93	55	37	Mature
28	18.88	53	37	Mature
29	40.81	56	39	Mature
30	46.72	53	37	Mature
31	61.20	56	37	Mature
32	33.92	53	38	Mature
33	59.32	82	37	Mature

a L/S ratio: lecithin/sphingomyelin ratio.

b LBC: lamellar body counts.

c GW: gestational week.

d FLM: fetal lung maturity.

**Table 2:** The clinical performance of L/S ratio and LBC in FLM prediction

N = 33	Premature	Mature	Sensitivity	Specificity
L/S < 10.0	10	0	91%	100%
L/S> 10.0	1	22		
LBC < 50 × 10^9^/L	8	1	73%	95%
LBC> 50 × 10^9^/L	3	21		

Note

FLM: fetal lung maturity; L/S: lecithin/sphingomyelin ratio; LBC: lamellar body counts.

### 3.3 The lipid metabolic profiling of amniotic fluid from different FLM and GWs

**Figure 2:**
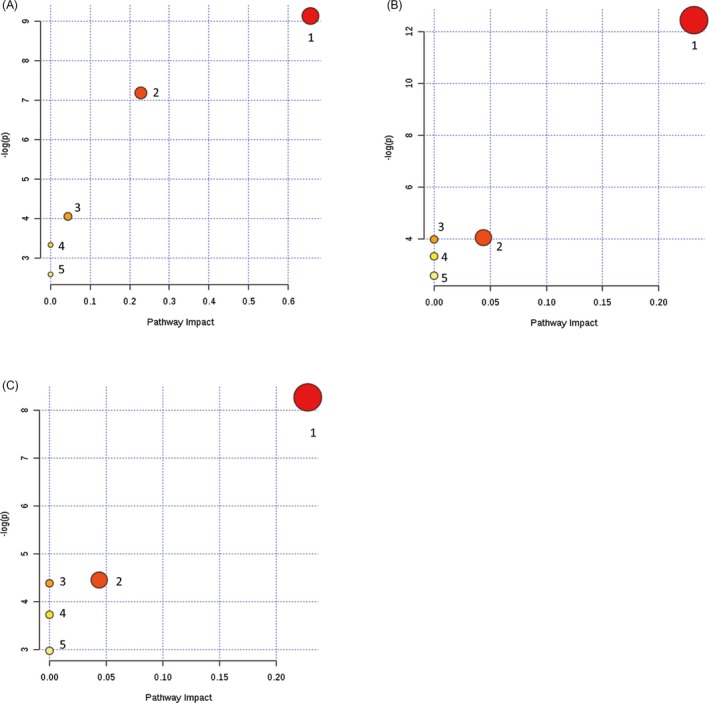
The pathway analysis of lipid metabolism among amniotic fluid of different FLM development status, presenting metabolic pathways arranged according to the scores from enrichment analysis (y‐axis) and from topology analysis (x‐axis). The darkness of the circles stands for the statistical significance in the corresponding pathway, and the radius represents the centrality measures. The pathway analysis details were elaborated in Table 4. A, comparison of lipid metabolism between premature group (GW < 37) and control group (GW = 18); B, comparison of lipid metabolism between mature group (GW < 37) and control group (GW = 18); C, comparison of lipid metabolism between mature group (GW> 38) and control group (GW = 18)

In the pathway enrichment analysis, the impact scores were a series of normalized results obtained from the pathway topology analysis, which indicate the location and importance of the significant metabolites in the corresponding metabolic pathways. Between the premature group (GW < 37) and control group (GW = 18), the most significant metabolic pathways in which the differently expressed lipids were located were listed in the order of decreasing impact scores: the linoleic acid metabolism (0.66), glycerophospholipid metabolism (0.23), glycosylphosphatidylinositol (GPI)‐anchor biosynthesis (0.04), alpha‐linolenic acid metabolism (0), and arachidonic acid metabolism (0). Interestingly, when the two mature groups of preterm (GW < 37) and term (GW > 38) newborns were compared with the control group (GW = 18) separately, the significantly different metabolic pathways with highest impact value were both identified as the glycerophospholipid metabolism (0.23) and the glycosylphosphatidylinositol (GPI)‐anchor biosynthesis (0.04). (Figure 2 and Table 3).

**Table 3:** Details for lipid metabolic pathway analysis in Figure 2

	Pathway name	‐Log(*p*)^a^	Impact^b^
Figure 2A	1‐Linoleic acid metabolism	9.1297	0.66
	2‐Glycerophospholipid metabolism	7.1824	0.23
	3‐Glycosylphosphatidylinositol (GPI)‐anchor biosynthesis	4.0539	0.04
	4‐Alpha‐linolenic acid metabolism	3.3319	0
	5‐Arachidonic acid metabolism	2.5859	0
Figure 2B	1‐Glycerophospholipid metabolism	12.445	0.23
	2‐Glycosylphosphatidylinositol (GPI)‐anchor biosynthesis	4.0539	0.04
	3‐Linoleic acid metabolism	3.9853	0
	4‐Alpha‐linolenic acid metabolism	3.3319	0
	5‐Arachidonic acid metabolism	2.5859	0
Figure 2C	1‐Glycerophospholipid metabolism	8.2707	0.23
	2‐Glycosylphosphatidylinositol (GPI)‐anchor biosynthesis	4.4566	0.04
	3‐Linoleic acid metabolism	4.3879	0
	4‐Alpha‐linolenic acid metabolism	3.7315	0
	5‐Arachidonic acid metabolism	2.9786	0

a ‐Log(*p*) represents levels of statistical significance of *p* value from pathway enrichment analysis.

b Impact represents the normalized pathway impact value from pathway topology analysis, which indicates the location and importance of the significant different metabolites in the corresponding metabolic pathway. (Note: The pathway with impact value 0 means a little association but cannot be neglected.)

### 3.4 Lipid biomarkers for predicting FLM in amniotic fluid

Four major lipid classes including PC, PE, SM, and Cer were detected and compared in the mature groups (GW < 37 or GW > 38) and the premature group (GW < 37). Of the top ten significant lipids identified, there were 8 PCs, 1 SM, and 1 PE of which the fold changes were ranged from 2.26 to 6.74.(Table 4).

**Table 4:** Top 10 significantly increased lipids in the fetal lung mature group

Compounds	Fold change (M/N)^a^	VIP value^b^	*P* value
PC(20:4/0:0)	6.7425	1.54297	0.000004729
PC(20:4/14:0)	4.4273	1.41996	0.000135970
PC(20:4/20:3)	3.3143	1.29499	0.000004357
PC(P‐18:0/18:1)	2.8585	1.14608	0.000380290
SM(d16:1/17:0)	2.7519	1.14195	0.000051375
PC(O‐16:0/18:1)	2.5012	1.2332	0.000005028
PC(P‐18:0/20:4)	2.4672	1.285	0.000000489
PC(16:1/14:0)	2.4554	1.12362	0.000184340
PE(16:0/16:0)	2.2939	1.25171	0.000117060
PC(18:2/19:0)	2.2626	1.38155	0.0000898380

a M/N: mature/premature.

b VIP value: variable importance for the projection value.

## 4 DISCUSSION

As the fetal lung contributes to the formation of the amniotic fluid, its composition may well reflect the stage of fetal lung development.^14^ The lung surfactants consist of approximately 90% lipids in which lecithin and PG are the most abundant (76%‐86% and 6%‐13%, respectively). The sphingomyelin accounts for approximately 2% of total lipids and keeps constant during the late pregnancy.^4^ The lecithin is synthesized from 28 GWs with a rapid rise around 36 GWs and continues to increase until delivery.^4^ The L/S ratio by TLC was the first biochemical test of assessing FLM development in the early 1970s and was considered as the gold standard in the past few decades.^4^ Nowadays, this testing method was much less commonly used in clinical laboratories due to the technical barriers of TLC that is often inaccurate, time‐consuming, and labor intensive.^5^ Alternatively, there had been some studies about the measurement of the L/S ratio by fast atomic bombardment mass spectrometry.^15^, ^16^ Later, Kwak and his colleagues established new methods to measure the L/S ratio on liquid chromatography‐tandem mass spectrometry (LC–MS/MS) and matrix‐assisted laser desorption and ionization time of flight mass spectrometry (MALDI‐TOF MS). These new MS‐based methods were comparable with the traditional TLC method in terms of the ability in FLM prediction.^12^, ^17^ In Kwak's study, the L/S ratio by mass spectrometry was calculated from the sum peak intensity of the six lecithin, and one peak intensity of sphingomyelin since these lipid peaks were the most abundant ones and easy to locate in the immature samples.^12^ In contrast, the LC‐HRMS applied in our study had better resolving power and was able to detect a comprehensive set of lipids including 64 lecithin and 23 sphingomyelins in the amniotic fluid samples. The same L/S ratio cutoff value of 10.0 that was determined in Kwak's article with an LC–MS/MS method was applied in our study with the LC‐HRMS method. It seemed to produce satisfactory distinguishing power in our FLM prediction study (Table 2), although larger patient cohort is warranted to further validate the cutoff values with the LC‐HRMS method.

The LBC has been proposed as a potential replacement of the L/S ratio since it can be simply performed on the platelet channel of hematological cell counters. Although some studies showed the LBC method performed well compared with the L/S ratio in FLM prediction, the laboratory‐specific cutoff values are yet to be established and the quality control reagents for LBC are not readily accessible.^4^ With the 33 amniotic fluid specimens of which the actual FLM status was evaluated by the Apgar scores, the sensitivity and specificity of LC‐HRMS determined L/S ratio were superior to those of the LBC method. Due to the limited number of the premature specimens available in this study, the efficiency of the L/S ratio method along with its cutoff value needs to be verified in larger sample groups.

As lipids play critical roles in fetal lung development,^4^ the targeted LC‐HRMS‐based lipidomic profiling approach was used to reveal the basic metabolic changes during the fetal lung maturation. When compared with the control group (GW = 18) which represents the early stage of lung development, the significantly different lipid metabolism pathways of different groups (premature vs mature) in our studies were essentially identical except for the linoleic acid metabolism, which was only enriched apparently in the premature group. Linoleic acid is a direct precursor of the bioactive oxidized linoleic acid metabolites and arachidonic acid, which produces pro‐inflammatory endocannabinoids and eicosanoids.^18^ Meanwhile, as part of structural membrane phospholipids, linoleic acid can maintain a certain degree of membrane fluidity of the transdermal water barrier of the epidermis,^19^ which may affect the exchange of air on the alveolar surface of the newborns. Additionally, a study conducted in Germany reported that the population with high intakes of palm oil had lower RDS incidence in preterm infants than the population using other oils, which may be related to the extremely high amounts of linoleic acid and palmitic acid in palm oil.^20^ Glycerophospholipid metabolism involves lecithin which is the most abundant lipid in amniotic fluid and changes rapidly during the late pregnancy as described before, so this may explain why glycerophospholipid metabolism was also found to be significant in fetal lung development.^21^ Furthermore, it was also reported that the arachidonic acid of sphingomyelin in human amniotic fluid showed an increase with gestational age although it has minimum impact in the fetal lung development with the amniotic fluid samples.^14^

To look for potential lipid biomarkers of predicting FLM and RDS in infants, the lipid components in amniotic fluid of 8 premature samples and 20 mature samples were analyzed using targeted LC‐HRMS‐based lipidomic profiling approach. Of the top ten most different lipids, the majority of the identified metabolites belonged to the PC, including PC(20:4/0:0), PC(20:4/14:0), PC(20:4/20:3), PC(P‐18:0/18:1), PC(O‐16:0/18:1), PC(P‐18:0/20:4), PC(16:1/14:0), and PC(18:2/19:0). In addition, the levels of SM (d16:1/17:0) and PE (16:0/16:0) were also different between the premature and the mature groups. All these different lipids were higher in the amniotic fluid samples with mature fetal lung development than those with premature lungs. To our best knowledge, few literatures were focused on the relationship of these lipid species and the pathogenesis of RDS. As lecithin is the main component in amniotic fluid, so it is understandable that the PCs accounted for the majority of the different lipids. A study indicated that PC(O‐16:0/18:1) is related to chlamydia trachomatis serovar D infectivity.^22^ Besides, PC(16:0/18:1) has been reported to be significantly increased in thyroid papillary and breast cancers compared with healthy controls.^23^, ^24^ However, as the studies of the above lipid candidates in FLM prediction are rather scarce, further studies with larger samples size are required to verify their clinical values in such area.

To decrease the incidence of RDS of preterm infants, lung promotion drugs such as corticosteroids are usually be given to mother before the planned delivery.^25^ Nevertheless, there may be long‐term adverse consequences of antenatal exposure to corticosteroids. Studies have shown that exposure to excess corticosteroids before birth may be linked to impaired fetal neurodevelopment, cardiovascular disease, and type 2 diabetes.^25^ Therefore, it is beneficial to timely and accurately predict FLM and avoid excessive pulmonary promotion drugs for mothers before delivery.

In this study, an efficient and accurate LC‐HRMS method was established to determine the L/S ratio in the amniotic fluid samples for the purpose of FLM prediction. The subsequent lipidomic profiling revealed the linoleic acid metabolism may play an important role in the early fetal lung development.
